# Five-Year Trajectories of Prescription Opioid Use

**DOI:** 10.1001/jamanetworkopen.2023.28159

**Published:** 2023-08-10

**Authors:** Natasa Gisev, Luke Buizen, Ria E. Hopkins, Andrea L. Schaffer, Benjamin Daniels, Chrianna Bharat, Timothy Dobbins, Sarah Larney, Fiona Blyth, David C. Currow, Andrew Wilson, Sallie-Anne Pearson, Louisa Degenhardt

**Affiliations:** 1 National Drug and Alcohol Research Centre, UNSW Sydney, Sydney, New South Wales, Australia; 2 School of Population Health, Faculty of Medicine and Health, UNSW Sydney, Sydney, New South Wales, Australia; 3 Bennett Institute for Applied Data Science, Nuffield Department of Primary Care Health Sciences, University of Oxford, Oxford, United Kingdom; 4 School of Population Health, UNSW Sydney, Sydney, New South Wales, Australia; 5 Department of Family Medicine and Emergency Medicine and Centre de Recherche du Centre Hospitalier de l’Université de Montréal, Université de Montréal, Montréal, Québec, Canada; 6 School of Public Health, Faculty of Medicine and Health, The University of Sydney, Sydney, New South Wales, Australia; 7 Faculty of Science, Medicine and Health, University of Wollongong, Wollongong, New South Wales, Australia; 8 Menzies Centre for Health Policy, Faculty of Medicine and Health, The University of Sydney, Sydney, New South Wales, Australia

## Abstract

**Importance:**

There are known risks of using opioids for extended periods. However, less is known about the long-term trajectories of opioid use following initiation.

**Objective:**

To identify 5-year trajectories of prescription opioid use, and to examine the characteristics of each trajectory group.

**Design, Setting, and Participants:**

This population-based cohort study conducted in New South Wales, Australia, linked national pharmaceutical claims data to 10 national and state data sets to determine sociodemographic characteristics, clinical characteristics, drug use, and health services use. The cohort included adult residents (aged ≥18 years) of New South Wales who initiated a prescription opioid between July 1, 2003, and December 31, 2018. Statistical analyses were conducted from February to September 2022.

**Exposure:**

Dispensing of a prescription opioid, with no evidence of opioid dispensing in the preceding 365 days, identified from pharmaceutical claims data.

**Main Outcomes and Measures:**

The main outcome was the trajectories of monthly opioid use over 60 months from opioid initiation. Group-based trajectory modeling was used to classify these trajectories. Linked health care data sets were used to examine characteristics of individuals in different trajectory groups.

**Results:**

Among 3 474 490 individuals who initiated a prescription opioid (1 831 230 females [52.7%]; mean [SD] age, 49.7 [19.3] years), 5 trajectories of long-term opioid use were identified: very low use (75.4%), low use (16.6%), moderate decreasing to low use (2.6%), low increasing to moderate use (2.6%), and sustained use (2.8%). Compared with individuals in the very low use trajectory group, those in the sustained use trajectory group were older (age ≥65 years: 22.0% vs 58.4%); had more comorbidities, including cancer (4.1% vs 22.2%); had increased health services contact, including hospital admissions (36.9% vs 51.6%); had higher use of psychotropic (16.4% vs 42.4%) and other analgesic drugs (22.9% vs 47.3%) prior to opioid initiation, and were initiated on stronger opioids (20.0% vs 50.2%).

**Conclusions and relevance:**

Results of this cohort study suggest that most individuals commencing treatment with prescription opioids had relatively low and time-limited exposure to opioids over a 5-year period. The small proportion of individuals with sustained or increasing use was older with more comorbidities and use of psychotropic and other analgesic drugs, likely reflecting a higher prevalence of pain and treatment needs in these individuals.

## Key Points

**Question:**

What are the 5-year trajectories of opioid use following initiation, and what are the characteristics of different trajectory groups?

**Findings:**

In this population-based cohort study of 3.47 million adults, 5 trajectories of opioid use were identified. Approximately 3% of individuals were classified to the sustained use trajectory group, which was characterized by individuals with older age, a higher number of comorbidities, and higher use of psychotropic and other analgesic drugs and health services vs other trajectory groups.

**Meaning:**

Findings from this study suggest that, although most individuals who commenced prescription opioid treatment had low, time-limited exposure to opioids, the small proportion of adults with sustained or increasing opioid use had greater clinical complexity and treatment needs.

## Teaser

This cohort study identifies 5-year trajectories of opioid use for acute pain and cancer pain among US adults.

## Introduction

Use of pharmaceutical opioids is widespread in Australia and many other countries.^1^ Approximately 3 million individuals in Australia receive dispensed opioids annually, and 1.9 million individuals in Australia initiate a prescription opioid in any given year.^2^ There are particular concerns about the long-term use of opioids, with known risks including potential dependence and increased mortality.^3,4,5^

Previous studies have attempted to quantify rates of long-term opioid use with several approaches and reported varied findings.^6,7,8^ Study populations are often heterogenous, excluding people with cancer or opioid use disorder and applying various definitions of long-term use that were determined a priori, resulting in a wide range of estimates of long-term use.^6,7,8^ A systematic review identified 78 definitions of long-term opioid use among 128 studies that used routinely collected data, with estimates of long-term opioid use prevalence in published studies ranging from 0.2% to 57%.^6^

Fewer studies have examined individual-level opioid use trajectories, and these studies have been conducted in select populations.^9,10,11,12^ Among individuals in Australia without cancer, 2.6% were identified to have 12-month trajectories indicating persistent use.^9^ In a study examining 4-year trajectories among US Medicaid enrollees aged 18 to 45 years, 6.0% of the cohort was classified as a sustained high use group.^10^ This small group accounted for 37% of all prescriptions filled and 61% of the dispensed oral morphine equivalent (OME) milligrams.^10^ A study from South Korea of 5-year trajectories among people with musculoskeletal disease found that a sustained high use group comprised 4.6% of the cohort.^11^ A 10-year trajectory model of self-reported opioid use data from older adults (aged ≥65 years) identified a prevalent chronic use group as consisting of 2.4% of the cohort.^12^ These studies were limited by the exclusion of important subpopulations, including people with cancer or nonmusculoskeletal pain,^9,11,12^ and age groups with chronic noncancer pain (CNCP), such as middle-aged and older adults,^10,12^ as well as the use of self-reported data of opioid use.^12^ There is a need to explore individual-level opioid use trajectories among the general population, which reflect opioid use in the clinical setting.

Understanding opioid use trajectories, and characteristics of individuals with these trajectories, is important to generate information to support prescribers and guide decision-making on opioid use policies in Australia and interventions to minimize future opioid-related harms and maximize treatment benefits. In this cohort study, called POPPY II, we aimed to identify 5-year trajectories of prescription opioid use after initiation for any indication among all residents of New South Wales, Australia, and to examine the characteristics of each trajectory group.

## Methods

### Study Design and Setting

POPPY II is a population-based cohort study of approximately 3.57 million adult residents in New South Wales, Australia, who initiated a prescription opioid between July 1, 2003, and December 31, 2018.^13,14^ The Australian Institute of Health and Welfare Ethics Committee, New South Wales Population and Health Services Research Committee, the Australian Capital Territory Health Human Research Ethics Committee, and the Australian Capital Territory Calvary Public Hospital Bruce Ethics Committee approved the study and waived the informed consent requirement to access deidentified linked personal health data. We followed the Strengthening the Reporting of Observational Studies in Epidemiology (STROBE) and Reporting of Studies Conducted Using Observational Routinely Collected Health Data Statement for Pharmacoepidemiology (RECORD-PE)^15^ reporting guidelines.

New South Wales is the most populous state in Australia, with approximately 7.9 million residents in 2018.^16^ Australia’s universal health care system provides subsidized health services, including subsidized drugs that are dispensed in the community under the Pharmaceutical Benefits Scheme (PBS).^17^ General beneficiaries receive PBS-listed drugs priced at less than or equal to a set maximum co-payment (A$39.50 [US$27.65] in 2018). Concessional beneficiaries (individuals who receive government assistance, including pensioners) pay a lower PBS co-payment (A$6.40 [US$4.50] in 2018).^18^ Prior to July 2012, available PBS data included dispensed PBS-listed drugs that attracted a subsidy, which were all drugs that were dispensed to concessional and general beneficiaries that cost more than the PBS co-payment. From July 2012, the data set includes all PBS-listed drugs for general beneficiaries.^17^

### The Cohort and Procedure

The cohort included adult residents (aged ≥18 years) of New South Wales who initiated a prescription opioid between July 1, 2003, and December 31, 2018, and who had at least 12 months of previous data and at least 2 months of follow-up beyond cohort entry (minimum requirements for trajectory analysis) (eFigure 1 in Supplement 1). Cohort entry was the date of index opioid dispensing, with no evidence of opioids dispensed in the previous 365 days. Observation began at cohort entry, and follow-up for each individual ended after 5 years, on the date of death, or on December 31, 2018, whichever occurred first. Opioids included were buprenorphine, codeine, dextropropoxyphene, fentanyl, hydromorphone, methadone, morphine, oxycodone, pethidine, tapentadol, and tramadol. Dispensed methadone and buprenorphine for the treatment of opioid dependence were not recorded in the PBS data set and were therefore not considered. The Anatomical Therapeutic Chemical (ATC) codes and PBS item codes used to derive the cohort are provided in eTable 1 in Supplement 1.

Prescriptions that were dispensed privately to an individual (for which the recipient pays the full cost) or to public hospital inpatients were not captured; these prescriptions represented a small proportion of overall opioid use in Australia.^19^ Extracted PBS records were linked to 10 Commonwealth and state data collections by the Australian Institute of Health and Welfare and the New South Wales Centre for Health Record Linkage using probabilistic linkage methods that incorporated personal identifiers (eg, name, age, date of birth, and address). eTable 2 in Supplement 1 provides a summary of linked data sets.

Evidence of a dispensed opioid prescription (yes or no) in each monthly interval was determined over the 60-month follow-up period, commencing from the end of the first month of observation. Individuals who exited the cohort early due to death or the end of the observation period had their subsequent opioid use set as missing in each of the remaining months. Consequently, at each monthly interval, the denominator used to determine the proportion of individuals using opioids was the total number of individuals remaining in the cohort at that time point.

### Baseline Characteristics

People’s month and year of birth were used to estimate age at cohort entry. Postcodes recorded at cohort entry were used to determine remoteness using the Australian Bureau of Statistics 2016 remoteness area classification system.^20,21^ Postcodes were also linked to the 2011 Index of Relative Socio-economic Disadvantage representing area-level economic and social resources (eg, household income, educational level, and employment status).^22^

Characteristics of the index opioid dispensing evaluated included number and type of opioid dispensed, route of administration, and prescriber type. Strong opioids were fentanyl, hydromorphone, morphine, oxycodone alone and oxycodone plus naloxone, and methadone or buprenorphine formulations for analgesia. The total OME milligrams of the index opioid dispensed were calculated by multiplying the strength and quantity of dispensed items by published conversion factors.^23^

Composite indicators were used to identify a range of common medical conditions in the 12 months prior to and including the day of cohort entry, incorporating dispensing history and contact with health services. Composite indicators were selected and developed on the basis of prior research and consultations with data custodians and both the research team and external clinical experts. Cancer registry notifications were used to identify cancer, and opioid agonist therapy registrations were used to ascertain opioid use disorder. People’s medical conditions and their corresponding *International Classification of Diseases, Ninth Revision*, *International Statistical Classification of Diseases, Tenth Revision*, and ATC codes^24,25^ are presented in eTable 3 in Supplement 1.

Use of health care services in the 12 months prior to and including the day of cohort entry was determined from records of primary care services, such as contact with general practitioners and allied health practitioners (eTable 4 in Supplement 1 shows item codes), and inpatient admissions, including emergency, nonemergency or planned, and other types. Use of nonopioid analgesics and psychotropic drugs that were dispensed in the 3 months prior to and including the day of cohort entry was also evaluated. Nonopioid analgesics included paracetamol, gabapentinoids, triptans, pizotifen, and nonsteroidal anti-inflammatory drugs. Psychotropic drugs included antiepileptics (excluding gabapentinoids), antipsychotics, anxiolytics, hypnotics and sedatives, and antidepressants. The ATC codes are presented in eTable 5 in Supplement 1.^25^

### Statistical Analysis

Group-based trajectory modeling was used to classify patterns of opioid use in the 60 months after cohort entry on the basis of monthly dispensing frequency. Trajectory modeling allows for the identification of homogenous subgroups in a population, with the number and composition of subpopulations derived from the data (ie, not defined a priori).^26,27^ A range of model specifications were considered, with varying trajectory orders (1-4) and number of groups (1-7) (eTable 6 and eFigure 2 in Supplement 1). The final model was selected using bayesian information criterion (BIC) and clinically relevant group allocations.^28^ Generally, BIC is used in trajectory model selection to identify the most parsimonious model that minimizes unexplained variation.^29^ As the BIC continued to improve with the addition of trajectory groups, the clinical meaningfulness of these additional trajectories was considered to facilitate the selection of the model with the smallest number of groups that represent distinct and plausible opioid use patterns. Using this process, a 5-group model was selected. Adequacy of the final model was determined on the basis of good correspondence between estimated and observed group membership probabilities and high average posterior probabilities of group membership.^27^ Characteristics of people in each trajectory group were compared descriptively. Analyses were conducted from February to September 2022, using SAS, version 9.4 (SAS Institute Inc).

Three sensitivity analyses were conducted. First, to assess the association of changes in ascertainment of under co-payment drugs (defined as drugs costing less than the government-set co-payment amount) that were dispensed after July 2012, the cohort was restricted to exclude general beneficiaries between 2002 and 2013 (ie, when there was incomplete capture of under co-payment drugs). The sensitivity analyses therefore included concessional beneficiaries who received an index opioid dispensed between July 2002 and December 2018 and general beneficiaries who received an index opioid dispensed between July 2013 and December 2018. Second, to assess the implications of people entering the cohort within 5 years of the data end date, the main analyses were replicated, but individuals who entered the cohort after 2014 were excluded. Third, to assess the implications of potential missing follow-up information, the main analyses were replicated, but all individuals with less than 5 years of follow-up for any reason including death were excluded. Person-level agreement to group allocations was assessed between the sensitivity and main analyses using the Cohen κ.^30^

## Results

Overall, 3 474 490 individuals met the criteria for inclusion. These individuals consisted of 1 831 230 females (52.7%) and 1 643 260 males (47.3%), with a mean (SD) age of 49.7 (19.3) years. Of this cohort, 26.0% were 65 years or older at cohort entry and 70.9% resided in major cities. During the follow-up period, 8.1% died.

### Trajectories of Opioid Use

Five trajectories of opioid use were identified based on dispensing frequency over 60 months: very low use (75.4%), low use (16.6%), moderate decreasing to low use (2.6%), low increasing to moderate use (2.6%), and sustained use (2.8%) (Figure). A heat map of the relative prevalence of key baseline characteristics and an overlaid histogram of age by each trajectory group are presented in eFigures 3 and 4 in Supplement 1.

**Figure.**
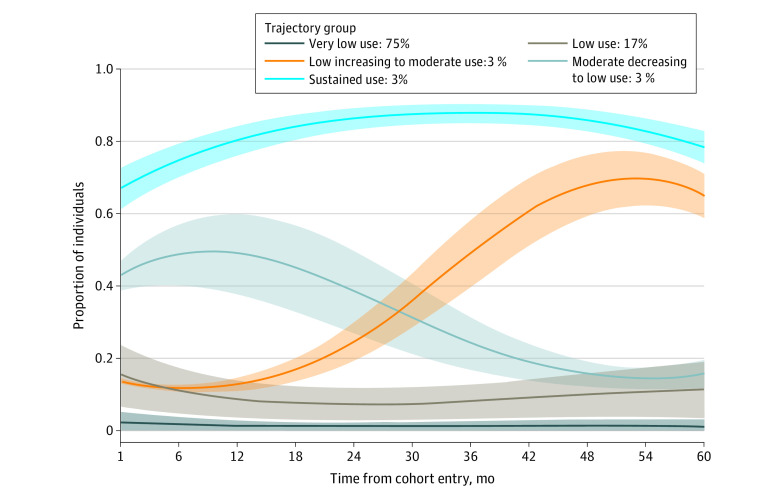
Five-Year Trajectories of Opioid Use in the cohort The solid lines represent the mean monthly proportion of the cohort using opioids in each trajectory group. The shaded areas indicate the 95% CIs.

### Patterns in Trajectory Groups

Overall, the very low use group comprised the largest percentage of younger individuals, while the sustained use group comprised the highest percentage of older people, with those 65 years or older at cohort entry representing 22.0% of the very low use group vs 58.4% of the sustained use group (Table 1). The sustained use group also had a lower percentage of individuals residing in major cities compared with the very low use group (62.4% vs 72.7%).

**Table 1.** Sociodemographic Characteristics at Cohort Entry by Trajectory Group

Characteristic	Trajectory group individuals, No. (%)				
	Sustained use (n = 96 035)	Moderate decreasing to low use (n = 90 828)	Low increasing to moderate use (n = 90 667)	Low use (n = 575 488)	Very low use (n = 2 621 472)
Sex					
Male	42 424 (44.2)	42 041 (46.3)	38 963 (43.0)	259 254 (45.0)	1 260 578 (48.1)
Female	53 611 (55.8)	48 787 (53.7)	51 704 (57.0)	316 234 (55.0)	1 360 894 (51.9)
Age, y					
18-24	1267 (1.3)	3309 (3.6)	3320 (3.7)	47 096 (8.2)	343 001 (13.1)
25-34	3500 (3.6)	6558 (7.2)	6180 (6.8)	65 860 (11.4)	440 524 (16.8)
35-44	6936 (7.2)	10 306 (11.3)	9558 (10.5)	77 348 (13.4)	449 095 (17.1)
45-54	11 764 (12.2)	13 875 (15.3)	12 785 (14.1)	85 956 (14.9)	407 816 (15.6)
55-64	16 450 (17.1)	18 357 (20.2)	17 177 (18.9)	107 839 (18.7)	404 766 (15.4)
65-74	17 578 (18.3)	17 426 (19.2)	18 089 (20.0)	103 176 (17.9)	344 083 (13.1)
75-84	20 837 (21.7)	14 149 (15.6)	16 280 (18.0)	64 487 (11.2)	174 618 (6.7)
≥85	17 703 (18.4)	6848 (7.5)	7278 (8.0)	23 725 (4.1)	57 569 (2.2)
Beneficiary type					
Concessional	73 231 (76.3)	66 822 (73.6)	68 982 (76.1)	380 737 (66.2)	1 236 629 (47.2)
General	22 804 (23.7)	24 006 (26.4)	21 685 (23.9)	194 751 (33.8)	1 384 843 (52.8)
Remoteness^a^^,^^b^					
Major city	59 527 (62.4)	60 128 (66.6)	57 883 (64.2)	396 650 (69.4)	1 890 851 (72.7)
Inner regional	27 007 (28.3)	22 432 (24.9)	24 196 (26.8)	130 518 (22.8)	541 603 (20.8)
Outer regional	8276 (8.7)	7067 (7.8)	7469 (8.3)	40 716 (7.1)	156 387 (6.0)
Remote or very remote	567 (0.6)	630 (0.7)	600 (0.7)	3769 (0.7)	13 230 (0.5)
Relative socioeconomic disadvantage^c^^,^^d^					
First quintile: most disadvantaged	17 779 (18.7)	19 611 (21.7)	18 655 (20.7)	122 042 (21.4)	459 576 (17.7)
Second quintile	20 205 (21.2)	18 794 (20.8)	19 249 (21.4)	112 836 (19.7)	457 938 (17.6)
Third quintile	23 167 (24.3)	21 722 (24.1)	22 373 (24.8)	135 153 (23.7)	596 512 (22.9)
Fourth quintile	17 940 (18.8)	16 732 (18.5)	16 618 (18.4)	109 836 (19.2)	528 990 (20.3)
Fifth quintile: least disadvantaged	16 230 (17.0)	13 348 (14.8)	13 231 (14.7)	91 547 (16.0)	557 668 (21.4)
Year of cohort entry					
2003-2006	27 112 (28.2)	31 484 (34.7)	33 175 (36.6)	175 907 (30.6)	484 272 (18.5)
2007-2009	20 205 (21.0)	14 982 (16.5)	17 927 (19.8)	82 009 (14.3)	253 822 (9.7)
2010-2012	22 220 (23.1)	19 643 (21.6)	18 281 (20.2)	114 677 (19.9)	410 359 (15.7)
2013-2015	15 154 (15.8)	14 978 (16.5)	16 252 (17.9)	134 205 (23.3)	813 673 (31.0)
2016-2018	11 344 (11.8)	9741 (10.7)	5032 (5.5)	68 690 (11.9)	659 346 (25.2)

^a^ Classified using the 2016 remoteness area indexes.^20^

^b^ Excludes 24 984 individuals with missing values for the overall cohort.

^c^ Classified using the 2011 Index of Relative Socio-economic Disadvantage.^22^

^d^ Excludes 26 738 individuals with missing values for the overall cohort.

Use of paracetamol plus codeine combinations at cohort entry was higher in the very low use group vs the sustained use group (64.8% vs 29.6%) (Table 2). The percentage of individuals receiving buprenorphine at cohort entry was more than 20-fold higher in the sustained use group than the very low use group (12.4% vs 0.6%). Use of transdermal formulations at cohort entry was highest in the sustained use group compared with the very low use group (16.5% vs 0.9%). The sustained use group also had higher OME milligrams dispensed at cohort entry. For example, 26.3% of individuals in the sustained use group were dispensed less than 100 OME milligrams vs 71.6% of those in the very low use group.

**Table 2.** Index Opioid Dispensing Characteristics at Cohort Entry by Trajectory Group

Characteristic	Trajectory group individuals, No. (%)				
	Sustained use (n = 96 035)	Moderate decreasing to low use (n = 90 828)	Low increasing to moderate use (n = 90 667)	Low use (n = 575 488)	Very low use (n = 2 621 472)
Opioid^a^					
Buprenorphine	11 953 (12.4)	3889 (4.3)	2356 (2.6)	9871 (1.7)	16 851 (0.6)
Codeine	1673 (1.7)	3412 (3.8)	4006 (4.4)	38 626 (6.7)	179 716 (6.9)
Dextropropoxyphene	121 (0.1)	122 (0.1)	131 (0.1)	473 (0.1)	701 (<0.1)
Fentanyl	3858 (4.0)	1217 (1.3)	915 (1.0)	3715 (0.6)	7027 (0.3)
Hydromorphone	321 (0.3)	154 (0.2)	72 (0.1)	502 (0.1)	1145 (<0.1)
Methadone	159 (0.2)	102 (0.1)	62 (0.1)	232 (<0.1)	532 (<0.1)
Morphine	5705 (5.9)	1784 (2.0)	1155 (1.3)	5749 (1.0)	11 210 (0.4)
Oxycodone	23 467 (24.4)	15 187 (16.7)	14 556 (16.1)	84 384 (14.7)	424 543 (16.2)
Oxycodone plus naloxone	3781 (3.9)	2278 (2.5)	1269 (1.4)	12 043 (2.1)	66 116 (2.5)
Paracetamol plus codeine	28 388 (29.6)	42 901 (47.2)	50 432 (55.6)	341 062 (59.3)	1 698 140 (64.8)
Pethidine^b^	36 (<0.1)	61 (0.1)	54 (0.1)	213 (<0.1)	539 (<0.1)
Tapentadol^c^	455 (0.5)	344 (0.4)	102 (0.1)	1329 (0.2)	7626 (0.3)
Tramadol	18 737 (19.5)	21 332 (23.5)	17 007 (18.8)	87 924 (15.3)	255 266 (9.7)
Opioid type^b^					
Strong opioid only	47 048 (49.0)	23 209 (25.6)	19 363 (21.4)	108 566 (18.9)	488 426 (18.6)
Other opioid only	47 817 (49.8)	66 509 (73.2)	70 421 (77.7)	459 896 (79.9)	2 095 839 (79.9)
Both strong and other opioids	1170 (1.2)	1110 (1.2)	883 (1.0)	7026 (1.2)	37 207 (1.4)
No. of opioids dispensed at index opioid dispensing^c^					
1	92 241 (96.1)	88 203 (97.1)	88 782 (97.9)	560 757 (97.4)	2 553 946 (97.4)
≥2	3794 (3.9)	2625 (2.9)	1885 (2.1)	14 731 (2.6)	67 626 (2.6)
Administration route^a^					
Oral	80 595 (83.9)	85 797 (94.5)	87 332 (96.3)	561 686 (97.6)	2 595 725 (99.0)
Transdermal	15 793 (16.5)	5104 (5.6)	3268 (3.6)	13 578 (2.4)	23 862 (0.9)
Parenteral injection	625 (0.7)	251 (0.3)	230 (0.3)	1269 (0.2)	3959 (0.2)
Other	46 (0.1)	15 (<0.1)	20 (<0.1)	67 (<0.1)	313 (<0.1)
Prescriber type					
Medical	94 682 (98.6)	88 790 (97.8)	87 391 (96.4)	545 907 (94.9)	2 352 842 (75.5)
Dental	746 (0.8)	1258 (1.4)	2388 (2.6)	22 267 (3.9)	223 204 (8.5)
Both medical and dental	75 (0.1)	27 (<0.1)	22 (<0.1)	245 (<0.1)	1850 (0.1)
Missing or other	532 (0.6)	753 (0.8)	866 (1.0)	7069 (1.2)	43 576 (1.7)
Total OME, mg					
<100	25 293 (26.3)	41 388 (45.6)	51 063 (56.3)	367 718 (63.9)	1 877 900 (71.6)
100-249	38 079 (39.7)	28 517 (31.4)	24 470 (27.0)	138 006 (24.0)	555 440 (21.2)
250-499	16 519 (17.2)	10 880 (12.0)	8448 (9.3)	40 800 (7.1)	117 266 (4.5)
500-749	5476 (5.7)	4146 (4.6)	2961 (3.3)	13 358 (2.3)	36 665 (1.4)
≥750	10 668 (11.1)	5897 (6.5)	3725 (4.1)	15 606 (2.7)	34 201 (1.3)

Abbreviation: OME, oral morphine equivalent.

^a^ Categories are not mutually exclusive.

^b^ Strong opioids are buprenorphine, fentanyl, hydromorphone, methadone, morphine, and oxycodone alone and oxycodone plus naloxone. Other opioids are codeine, dextropropoxyphene, pethidine, tapentadol, and tramadol.

^c^ Accounts for different types of opioids only and not different formulations and strengths of the same opioid.

For most medical conditions, the percentage of individuals with each condition in the 12 months prior to opioid initiation was highest in the sustained use group (Table 3). The percentage of individuals with cancer was 5.4-times higher (22.2% vs 4.1%), dementia was 14-times higher (7.0% vs 0.5%), and depression was twice as high (30.8% vs 14.0%) in the sustained use group vs the very low use group. The percentage of individuals with opioid use disorder and other substance use disorder were highest in the moderate decreasing to low use group (0.9% and 3.6%) and low increasing to moderate use group (0.8% and 3.7%) compared with other trajectory groups.

**Table 3.** Evidence of Diagnoses or Treatment for Medical Conditions in the 12 Months Prior to Cohort Entry by Trajectory Group^a^

Condition	Trajectory group individuals, No. (%)				
	Sustained use (n = 96 035)	Moderate decreasing to low use (n = 90 828)	Low increasing to moderate use (n = 90 667)	Low use (n = 575 488)	Very low use (n = 2 621 472)
Cardiovascular					
Arrhythmia	6245 (6.5)	3753 (4.1)	4023 (4.4)	16 311 (2.8)	38 734 (1.5)
CHF	14 483 (15.1)	8755 (9.6)	9066 (10.0)	33 479 (5.8)	68 534 (2.6)
Hyperlipidemia	30 674 (31.9)	29 026 (32.0)	30 160 (33.3)	154 469 (26.8)	466 752 (17.8)
Hypertension	26 961 (28.1)	21 782 (24.0)	23 520 (25.9)	104 150 (18.1)	266 422 (10.2)
Ischemic heart disease	10 247 (10.7)	7731 (8.5)	8269 (9.1)	33 470 (5.8)	74 466 (2.8)
Endocrine					
Diabetes	13 152 (13.7)	12 186 (13.4)	12 210 (13.5)	64 268 (11.2)	183 064 (7.0)
Hypothyroidism	6454 (6.7)	5276 (5.8)	5831 (6.4)	28 208 (4.9)	96 802 (3.7)
Hyperthyroidism	495 (0.5)	322 (0.4)	294 (0.3)	1737 (0.3)	6412 (0.2)
Mental and Neurological					
Anxiety	13 771 (14.3)	13 635 (15.0)	13 776 (15.2)	61 444 (10.7)	152 724 (5.8)
Dementia	6740 (7.0)	1854 (2.0)	1579 (1.7)	5296 (0.9)	12 669 (0.5)
Depression	29 544 (30.8)	27 023 (29.8)	26 758 (29.5)	131 988 (22.9)	365 918 (14.0)
OUD	594 (0.6)	773 (0.9)	707 (0.8)	3480 (0.6)	8011 (0.3)
Other SUD	3018 (3.1)	3271 (3.6)	3369 (3.7)	18 116 (3.1)	51 937 (2.0)
Parkinson disease	3149 (3.3)	1700 (1.9)	1742 (1.9)	6090 (1.1)	14 200 (0.5)
Psychoses	9313 (9.7)	4363 (4.8)	4056 (4.5)	18 353 (3.2)	50 473 (1.9)
Musculoskeletal					
Osteoporosis	9391 (9.8)	6589 (7.3)	7506 (8.3)	27 678 (4.8)	60 763 (2.3)
Rheumatic disease	4369 (4.5)	3481 (3.8)	3746 (4.1)	15 807 (2.7)	41 773 (1.6)
Respiratory					
Asthma	14 102 (14.7)	13 280 (14.6)	14 396 (15.9)	74 354 (12.9)	223 539 (8.5)
COPD	7611 (7.9)	5039 (5.5)	5404 (6.0)	20 696 (3.6)	43 346 (1.7)
Other					
Cancer	21 364 (22.2)	9195 (10.1)	8538 (9.4)	38 458 (6.7)	107 001 (4.1)
Chronic liver failure	3773 (3.9)	2032 (2.2)	1863 (2.1)	7851 (1.4)	18 790 (0.7)
Hepatitis C	636 (0.7)	543 (0.6)	519 (0.6)	2318 (0.4)	5054 (0.2)
HIV/AIDS	117 (0.1)	76 (0.1)	73 (0.1)	663 (0.1)	3743 (0.1)
Kidney disease	3485 (3.6)	2004 (2.2)	1861 (2.1)	7958 (1.4)	17 260 (0.7)

Abbreviations: CHF, congestive heart failure; COPD, chronic obstructive pulmonary disease; OUD, opioid use disorder; SUD, substance use disorder.

^a^ A composite indicator for each medical condition was derived using information from each individual’s Pharmaceutical Benefits Scheme dispensing history, contact with inpatient hospital services, and contact with community mental health services. Additionally, these data sets were used in conjunction with cancer registry notifications to identify cancer and registrations for opioid agonist therapy treatment to identify OUD (eTable 3 in Supplement 1).

The percentage of individuals accessing an allied health practitioner in the 12 months prior to cohort entry was highest in the sustained use group and lowest in the very low use group (12.7% vs 8.2%) (Table 4). Similarly, the percentage of individuals with an inpatient admission was highest in the sustained use group and lowest in the very low use group (51.6% vs 36.9%).

**Table 4.** Health Service Use and Analgesic and Psychotropic Drugs Dispensed Prior to Cohort Entry by Trajectory Group

	Trajectory group individuals, No. (%)				
	Sustained use (n = 96 035)	Moderate decreasing to low use (n = 90 828)	Low increasing to moderate use (n = 90 667)	Low use (n = 575 488)	Very low use (n = 2 621 472)
**Health service use in the 12 mo prior to cohort entry**					
General or allied health services	89 700 (93.4)	85 843 (94.5)	85 245 (94.0)	550 196 (95.6)	2 490 630 (95.0)
GP visit	89 629 (93.3)	85 807 (94.5)	85 216 (94.0)	549 991 (95.6)	2 489 331 (95.0)
AHP visits					
Any AHP	12 206 (12.7)	10 751 (11.8)	9529 (10.5)	57 759 (10.0)	214 708 (8.2)
Chiropractor	464 (0.5)	526 (0.6)	422 (0.5)	2629 (0.5)	9937 (0.4)
Exercise physiologist	438 (0.5)	478 (0.5)	428 (0.5)	2881 (0.5)	10 835 (0.4)
Osteopathy	196 (0.2)	214 (0.2)	171 (0.2)	1061 (0.2)	3728 (0.1)
Physiotherapist	2986 (3.1)	3344 (3.7)	2721 (3.0)	17 517 (3.0)	57 345 (2.2)
Podiatry	7075 (7.4)	4291 (4.7)	4027 (4.4)	18 817 (3.3)	55 109 (2.1)
Psychologist	2153 (2.2)	3055 (3.4)	2701 (3.0)	20 671 (3.6)	95 437 (3.6)
Hospital admissions^a^					
Any admission	49 553 (51.6)	37 161 (40.9)	37 917 (41.8)	218 525 (38.0)	966 745 (36.9)
Emergency	33 373 (34.8)	20 973 (23.1)	19 315 (21.3)	100 950 (17.5)	335 939 (12.8)
Nonemergency or planned	28 544 (29.7)	23 242 (25.6)	25 108 (27.7)	145 495 (25.3)	690 399 (26.3)
Other	10 037 (10.5)	5346 (5.9)	4544 (5.0)	26 483 (4.6)	102 601 (3.9)
**Analgesic and psychotropic drugs dispensed in the 3 mo prior to cohort entry**					
Any analgesic	45 420 (47.3)	40 661 (44.8)	38 959 (43.0)	203 645 (35.4)	600 032 (22.9)
Paracetamol	30 044 (31.3)	20 112 (22.1)	19 382 (21.4)	80 460 (14.0)	165 830 (6.3)
Pregabalin	1763 (1.8)	1451 (1.6)	738 (0.8)	5816 (1.0)	18 557 (0.7)
Gabapentin	517 (0.5)	312 (0.3)	310 (0.3)	953 (0.2)	1730 (0.1)
Triptans	473 (0.5)	699 (0.8)	652 (0.7)	4150 (0.7)	13 014 (0.5)
Pizotifen	280 (0.3)	370 (0.4)	386 (0.4)	1707 (0.3)	3676 (0.1)
Any NSAID	21 412 (22.3)	26 558 (29.2)	25 656 (28.3)	144 928 (25.2)	461 657 (17.6)
Nonselective NSAIDs^b^	8057 (8.4)	10 860 (12.0)	10 703 (11.8)	68 043 (11.8)	247 039 (9.4)
Selective COX-2 inhibitors^c^	14 409 (15.0)	17 248 (19.0)	16 231 (17.9)	84 083 (14.6)	229 729 (8.8)
Any psychotropic medicine^d^	40 715 (42.4)	34 319 (37.8)	33 857 (37.3)	158 001 (27.5)	430 540 (16.4)
Antidepressants	17 458 (18.2)	15 945 (17.6)	16 027 (17.7)	74 213 (12.9)	201 922 (7.7)
Antiepileptics^e^	4826 (5.0)	3101 (3.4)	2955 (3.3)	13 519 (2.3)	35 964 (1.4)
Antipsychotics	13 553 (14.1)	8845 (9.7)	8192 (9.0)	38 320 (6.7)	106 172 (4.1)
Anxiolytics	9534 (9.9)	9363 (10.3)	9389 (10.4)	39 294 (6.8)	95 915 (3.7)
Hypnotics or sedatives	11 224 (11.7)	8596 (9.5)	8628 (9.5)	33 520 (5.8)	71 013 (2.7)

Abbreviations: AHP, allied health practitioner; COX, cyclooxygenase; GP, general practitioner; NSAID, nonsteroidal anti-inflammatory drug.

^a^ For individuals with multiple admission types in the 12 months prior to cohort entry.

^b^ Includes diclofenac, ibuprofen, indomethacin, ketoprofen, mefenamic acid, naproxen, piroxicam, and sulindac.

^c^ Includes celecoxib, lumiracoxib, meloxicam, and rofecoxib.

^d^ Classified according to World Health Organization Anatomical Therapeutic Chemical categories.

^e^ Excludes pregabalin and gabapentin.

The percentage of individuals with other analgesic use in the 3 months prior to study entry was twice as high in the sustained use group as in the very low use group (47.3% vs 22.9%) (Table 4). The percentage of individuals in the sustained group with psychotropic use was also 2.5-times higher compared with the very low use group (42.4% vs 16.4%). Past 3-month use of nonsteroidal anti-inflammatory drugs was highest in the moderate decreasing to low use group and the low increasing to moderate use groups (29.2% and 28.3%).

### Sensitivity Analysis

Trajectory modeling was repeated to ascertain whether the differences in cohort entry requirements were factors in changing opioid use patterns and allocation to trajectory groups and to assess the implications of missing data if individuals entered the cohort within 5 years of the follow-up end date or due to death. All trajectory group memberships and associated characteristics were consistent with those in the main analysis, with a high level of agreement in group allocations (Cohen κ range: 0.93-0.99) (eTable 7 and eFigure 5 in Supplement 1).^30^

## Discussion

In this study of 3 474 490 individuals initiating an opioid medicine, 5 distinct 5-year opioid use trajectories following the index opioid dispensing were identified. Most of the cohort (92.0%) had very low or low opioid use. Individuals with sustained use or low increasing to moderate use were older, had more comorbidities, and had higher use of other drugs recorded in the period before opioid initiation.

To our knowledge, this is the first published study examining opioid use trajectories over 12 months or longer in adults with or without cancer. In studies using trajectory modeling to identify opioid use patterns among select populations, similarly small, sustained use groups were identified. For example, in studies excluding people with cancer or limiting participation by age, between 2.4% and 6.0% of cohort members were classified under the sustained use group.^9,10,11,12^ The similarity in groups identified in these international studies suggests that the opioid trajectories identified and demographic characteristics of trajectory groups are generalizable to populations in high-income countries outside Australia with similar health and social systems.^9,10,11,12^ Overall, these findings suggest that most people who initiate an opioid prescription are likely to have low, time-limited exposure to opioids with little indication of ongoing use. This possibility is an important consideration for policymakers and stakeholders considering population-level prescribing of high-risk drugs. Opioids are essential drugs for acute and cancer pain, and many people with CNCP benefit from opioids.^31^ Continued focus and policy responses based on findings from a small group of people with increased risk of harms run the risk of limiting access to people who safely derive objective benefits from opioids.

The sustained use, low increasing to moderate use, and moderate decreasing to low use trajectory groups appeared to include people with complex needs. The characteristics observed in these groups are consistent with those in previous work and resemble the factors associated with dose escalation and transition to stronger opioids.^9,32^ These characteristics included older age,^9,11,12,33^ comorbid depression or psychotic illness,^9,11,33^ and use of psychotropics and nonopioid analgesics.^9,11,12,33^ Previous research has highlighted both the sociodemographic and clinical complexities of people with CNCP, including those initiating opioid therapy.^34,35,36,37^ The characteristics of the index opioid dispensing observed in the sustained use group are also concordant with those in the literature, with prior research demonstrating that individuals with persistent use were more likely to receive higher index OME milligrams^9,33^ or to initiate a strong opioid or transdermal formulation.^9^ Higher rates of cancer were also identified among the sustained use group: opioids were commonly prescribed along with evidence-based analgesics to treat severe pain associated with cancer.^38,39^ Overall, these findings suggest that most of the cohort received opioids for a short period, in keeping with clinical presentations that require temporary use of opioids, with the sustained use group including more people with evidence of health conditions for which opioid treatment may be appropriate, such as cancer.

Depression was highly prevalent in the sustained use, low increasing to moderate use, and moderate decreasing to low use trajectory groups. Associations between CNCP and depression have been found,^34^ which may explain the higher rate of depression in these groups and further highlight the complex needs of this population. Prior research has also identified almost twice the hazard of transition to long-term opioid use among people diagnosed with depression and people who were prescribed antidepressant medications.^40^ However, in the absence of indication information, antidepressants that were dispensed for other indications may have been misclassified as drugs that were dispensed for depression. Several antidepressant classes are indicated for pain conditions, and in a previous study of Australians taking opioids for CNCP, the most commonly reported antidepressants had pain indications (eg, amitriptyline).^41^ Higher rates of dementia were identified in the sustained use group, potentially due to the older age of this group; these results support previous findings that dementia may be associated with long-term opioid use.^33^ Conversely, at least 1 previous study has identified an inverse association between dementia and persistent opioid use,^12^ suggesting the need for further exploration.

Although the outcomes associated with trajectory group membership were not examined, there is evidence that trajectories with higher opioid use may be associated with an increased risk of harms. A study of people 65 years or older with CNCP and new opioid use found that individuals with increasing or consistent opioid dose trajectories had a higher risk of opioid-related adverse events than those with decreasing opioid dose trajectories.^42^ Similarly, studies examining opioid use among US veterans identified higher risk of opioid-related adverse events, hospitalization, and all-cause mortality among high use and increasing use groups.^43,44^ Improved understanding of the characteristics of individuals with higher use trajectories could benefit clinicians who initiate opioid therapy, providing information to support monitoring and interventions to prevent future problems as well as assisting with improved targeting of resource-limited interventions where they are most likely to be of benefit.

### Strengths and Limitations

Inclusion of all individuals with a newly dispensed opioid on the PBS allowed the exploration of opioid trajectories among all residents in New South Wales, Australia, over a 15-year period. The data-driven approach of group-based trajectory modeling allowed long-term opioid use to be defined by dispensing patterns rather than an a priori definition of long-term use.

Study findings should be interpreted in light of several limitations. During the study, low-dose formulations of codeine were available without prescription, but this was not captured in this data set, nor were opioids dispensed through private prescriptions or administered in public hospitals. As noted, the latter represented a minority of opioid prescribing in Australia and its implications for the findings of the present study are expected to be minimal.^19^ Sensitivity analyses were conducted to limit issues from the undercapture of prescriptions dispensed to general beneficiaries before 2012 and to assess the implications of missing data. Available data sets did not provide information about indication, dose, or intended treatment duration of opioids, and dispensed quantities were not examined. The relatively crude exposure measure (evidence of ≥1 opioid dispensed each month) did not account for whether people actually used dispensed opioids or whether time-dependent factors were accounted for. This descriptive study did not aim to explore causal factors, and independent factors associated with long-term opioid use trajectories were not assessed.

## Conclusions

In this population-based cohort study, distinct 5-year trajectories of opioid use were identified. Although most individuals commencing opioid use had relatively low opioid use over a 5-year period, 1 in 10 individuals demonstrated sustained or moderate opioid use. These individuals were older and had more comorbidities and use of psychotropic and other analgesic drugs, which likely reflected a higher prevalence of pain and treatment needs in these individuals. Information on the individual characteristics of people with different trajectories of opioid use may be used to prevent future harms from long-term opioid use through targeted monitoring and interventions. This study demonstrated the benefits of using data-driven approaches to examine individual patterns and trajectories of opioid use.

## Notes

Supplement 1. **eFigure 1.** Study Flow Diagram **eTable 1.** ATC and PBS Codes Used to Define the Cohort **eTable 2.** Detailed Description of Datasets Included in the Linkage **eTable 3.** ICD-10 and ATC Codes Used to Identify Medical Conditions of Interest **eTable 4.** MBS Item Codes for Primary Care, Allied Health, and Specialist Services **eTable 5.** ATC Codes for Non-Opioid Analgesic and Psychotropic Medicines **eTable 6.** Model Selection - BIC of Models With Varying Number of Trajectory Groups and Polynomial Order **eFigure 2.** Model Selection - Trajectory Plots of Models With Varying Opioid Use Groups **eFigure 3.** Heat Map Showing the Relative Prevalence of Baseline Characteristics by Trajectory Group **eFigure 4.** Overlaid Histograms of Age by Trajectory Group **eTable 7.** Comparison of Trajectory Group Membership for the Main and Sensitivity Analyses **eFigure 5.** Trajectory Plots for Sensitivity Analyses

Supplement 2.
Data Sharing Statement
