# Supplementary material for: Five-Year Trajectories of Prescription Opioid Use
Source: JAMA Netw Open. 2023 Aug 10;6(8):e2328159. doi: 10.1001/jamanetworkopen.2023.28159 (PMC10415961; doi:10.1001/jamanetworkopen.2023.28159)
Supplement: Supplement 1. — eFigure 1. Study Flow Diagram eTable 1. ATC and PBS Codes Used to Define the Cohort eTable 2. Detailed Description of Datasets Included in the Linkage eTable 3. ICD-10 and ATC Codes Used to Identify Medical Conditions of Interest eTable 4. MBS Item Codes for Primary Care, Allied Health, and Specialist Services eTable 5. ATC Codes for Non-Opioid Analgesic and Psychotropic Medicines eTable 6. Model Selection - BIC of Models With Varying Number of Trajectory Groups and Polynomial Order eFigure 2. Model Selection - Trajectory Plots of Models With Varying Opioid Use Groups eFigure 3. Heat Map Showing the Relative Prevalence of Baseline Characteristics by Trajectory Group eFigure 4. Overlaid Histograms of Age by Trajectory Group eTable 7. Comparison of Trajectory Group Membership for the Main and Sensitivity Analyses eFigure 5. Trajectory Plots for Sensitivity Analyses [file jamanetwopen-e2328159-s001.pdf]

Supplemental Online Content

Gisev N, Buizen L, Hopkins RE, et al. Five-year trajectories of prescription opioid use. *JAMA Netw Open*. 2023;6(8):e2328159.
doi:10.1001/jamanetworkopen.2023.28159

eFigure 1. Study Flow Diagram

eTable 1. ATC and PBS Codes Used to Define the Cohort

eTable 2. Detailed Description of Datasets Included in the Linkage

eTable 3. ICD-10 and ATC Codes Used to Identify Medical Conditions of Interest

eTable 4. MBS Item Codes for Primary Care, Allied Health, and Specialist Services

eTable 5. ATC Codes for Non-Opioid Analgesic and Psychotropic Medicines

eTable 6. Model Selection - BIC of Models With Varying Number of Trajectory Groups and Polynomial Order

eFigure 2. Model Selection - Trajectory Plots of Models With Varying Opioid Use Groups

eFigure 3. Heat Map Showing the Relative Prevalence of Baseline Characteristics by Trajectory Group

eFigure 4. Overlaid Histograms of Age by Trajectory Group

eTable 7. Comparison of Trajectory Group Membership for the Main and Sensitivity Analyses

eFigure 5. Trajectory Plots for Sensitivity Analyses

This supplemental material has been provided by the authors to give readers additional information about their work.

© 2023 Gisev N et al. *JAMA Network Open.*

eFigure 1. Study Flow Diagram

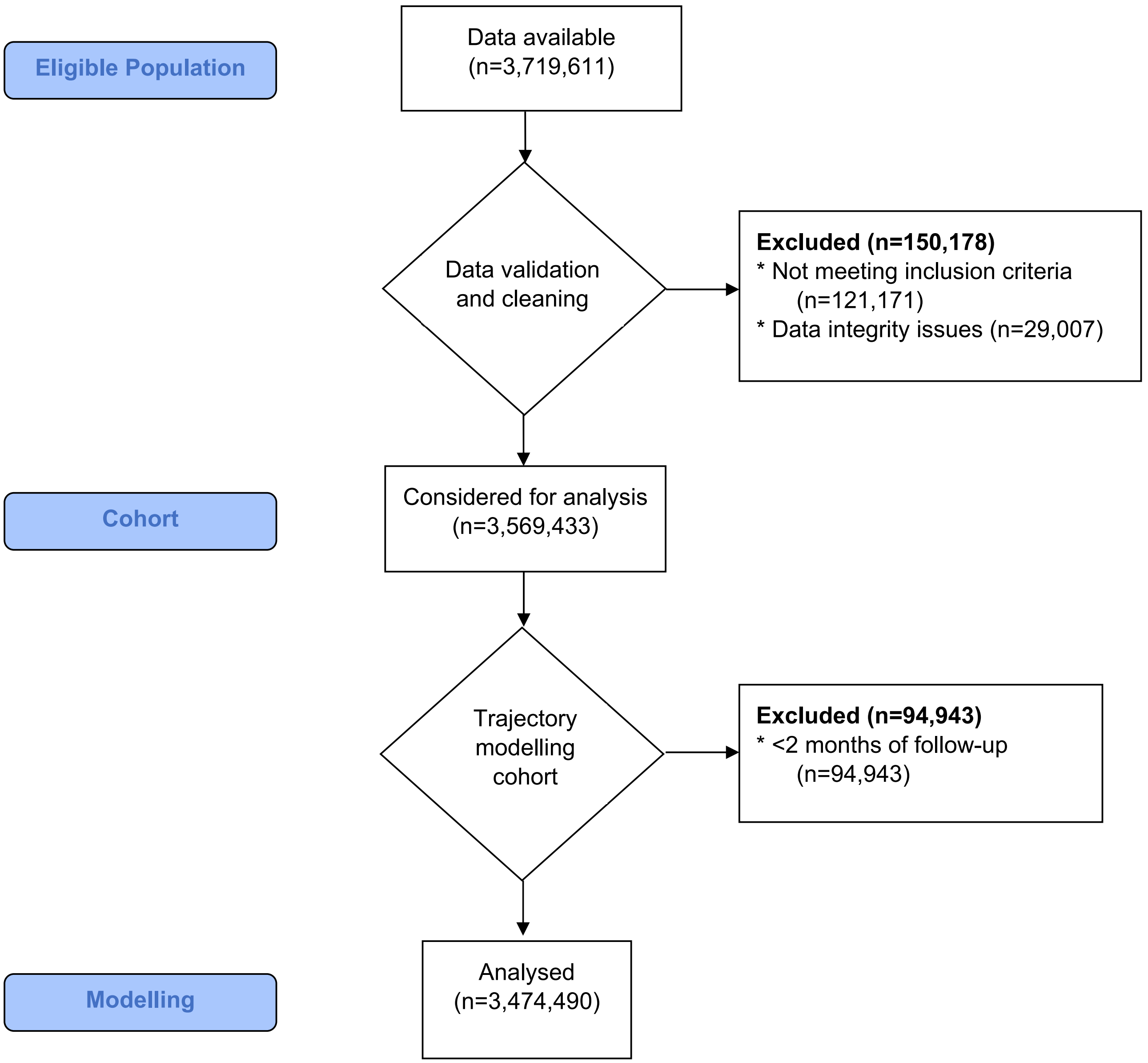

© 2023 Gisev N et al. *JAMA Network Open.*

eTable 1. ATC and PBS Codes Used to Define the Cohort

Opioid	Classification	ATC code[a]	PBS item code
**Buprenorphine**	Strong	N02AE01	08865N, 08866P, 08867Q, 10746N, 10755C, 10756D, 10770W, 10948F, 10949G, 10953L, 10957Q, 10959T, 10964C, 10970J, 06097X, 06098Y, 07201B
**Codeine**	Other	N02BE51<br>N02AA59<br>R05DA04<br>N02AJ06<br>N02AJ07<br>N02BA51<br>N02AA	04061R, 01214X, 05063L, 04286N, 01215Y, 03316M, 04170L, 04171M, 04275B, 08785J, 10186D, 07375E, 07530H, 06031K, 06032L
**Dextropropoxyphene**	Other	N02AC04	04081T
**Fentanyl**	Strong	N02AB03	08337T, 08338W, 08339X, 08340Y, 05413X, 05414Y, 05415B, 05416C, 05417D, 05418E, 05265D, 05277R, 05278T, 05279W, 05280X, 05401G, 05402H, 05403J, 05404K, 05405L, 05406M, 05407N, 05408P, 05409Q, 05410R, 05411T, 05412W, 05437E, 05438F, 05439G, 05440H, 05441J, 08878G, 08891Y, 08892B, 08893C, 08894D, 10600X, 10601Y, 10602B, 10603C, 10604D, 10606F, 10607G, 10608H, 10610K, 10611L, 10612M, 10613N, 10684H, 10697B, 10698C, 10713W, 10722H, 10723J, 10729Q, 10737D, 10738E, 10739F, 07202C, 07203D, 07204E, 07205F, 07206G
**Hydromorphone**	Strong	N02AA03	03357Q, 03358R, 03367F, 03368G, 05023J, 05129Y, 05130B, 05131C, 08423H, 08422G, 05115F, 05116G, 05117H, 05132D, 08420E, 08421F, 08424J, 08541M, 08542N, 08543P, 09299K, 09406C, 09407D, 09408E, 09409F, 06062C, 06075R, 06076T, 06077W, 07217W, 07218X, 07219Y, 07220B, 07221C
**Methadone**	Strong	N02AC52<br>N02AC	01606M, 01609Q, 05399E, 05400F, 06035P, 06036Q, 06037R
**Morphine**	Strong	N02AA01	05253L, 05254M, 05255N, 05256P, 09014K, 09015L, 09016M, 09017N, 05064M, 05065N, 05066P, 05067Q, 05161P, 05162Q, 05164T, 05165W, 05166X, 05167Y, 05171E, 05240T, 05241W, 05242X, 05243Y, 05244B, 05245C, 05246D, 02332R, 02333T, 05392T, 05395Y, 05396B, 01607N, 01644M, 01645N, 01646P, 01647Q, 01653B, 01654C, 01655D, 01656E, 02122Q, 02123R, 02124T, 02839K, 02840L, 02841M, 03479D, 03480E, 04349X, 05163R, 05168B, 05169C,

© 2023 Gisev N et al. *JAMA Network Open.*

			05170D, 05237P, 05238Q, 05239R, 05391R, 05393W, 05394X, 08035X,
**Opioid**	**Classification**	**ATC code[a]**	**PBS item code**
**Morphine (continued)**	Strong	N02AA01	08146R, 08305D, 08306E, 08349K, 08453X, 08454Y, 08489T, 08490W, 08491X, 08492Y, 08493B, 08494C, 08669G, 08670H, 10858L, 10862Q, 10863R, 10864T, 10868B, 10869C, 10874H, 10878M, 07909G, 07910H, 06047G, 06048H, 06049J, 07207H, 07208J, 07209K, 07210L, 06038T, 06039W, 06040X, 06041Y, 06042B, 06050K, 06051L, 06052M, 06053N, 06054P, 06055Q, 06056R, 06057T, 06064E, 06065F, 06066G, 06067H, 06068J, 06069K, 06099B
**Oxycodone**	Strong	N02AA05	05015Y, 05016B, 05198N, 02481N 02622B 05190E, 05119F, 05194J, 05195K, 05197M, 05227D, 05247E, 05248F, 05249G, 05250H, 08000C, 08385H, 08386J, 08387K, 08388L, 08464L, 08501K, 08502L, 08644Y, 08681X, 09399Q, 09400R,
**Oxycodone/Naloxone**	Strong	N02AA55	08934F, 08935G, 08936H, 10757E, 10758F, 10776E, 11102H, 11111T
**Pethidine**	Other	N02AB02	01828F, 03483H, 05199P, 01829G, 05200Q, 06045E, 06046F
**Tapentadol**	Other	N02AX06	10091D, 10092E, 10094G, 10096J, 10100N
**Tramadol**	Other	N02AX02	03338Q, 05001F, 05002G, 05003H, 05234L, 05235M, 05236N, 09199E, 09200F, 09201G, 02527B, 03484J, 05150C, 05231H, 05232J, 08455B, 08523N, 08524P, 08525Q, 08582Q, 08611F, 08843K, 06072N, 06073P, 06074Q, 06096W, 07211M, 07212N, 07213P, 07214Q

ATC: Anatomical Therapeutic Chemical classification; PBS: Pharmaceutical Benefits Scheme
[a] Includes non-WHO codes used by the PBS data custodian

© 2023 Gisev N et al. *JAMA Network Open.*

eTable 2. Detailed Description of Datasets Included in the Linkage

Dataset name and date range	Description of dataset	Purpose of dataset	Key variables of interest[a]
Pharmaceutical Benefits Scheme (PBS), 01/07/2002 - 31/12/2018	Records for all PBS-listed medicines for which the Commonwealth pays a subsidy (2002-2012). After 2012, all PBS dispensings are included.	To identify the cohort and the types of opioids and other medicines dispensed.	PBS-item number, date of prescribing and dispensing, patient/pharmacy/prescriber postcodes, patient co-payment amount, cost to government, provider location.
Medicare Enrolment File (MEF), 01/01/2002 - 31/12/2018	Contains Medicare enrolment details (e.g. name, address history, date of birth). Used by the Australian Institute of Health and Welfare to identify individuals and link records across datasets.	To provide month/year of birth and sex information for all cohort members.	Month and year of birth, sex.
Medicare Benefits Scheme (MBS), 01/01/2002 - 31/12/2018	Claims for all medical and hospital services subsidised by the Commonwealth including doctor visits, allied health, pathology tests and imaging.	To identify the use of medical and hospital services.	MBS-item number, date of service, schedule fee, provider charge, benefit paid, patient co-payment, provider location.
Australian Cancer Database (ACD), 01/01/1982 - 31/12/2019	All notifications of primary malignant neoplasms.	To identify individuals potentially treated with opioids for cancer pain.	Date of diagnosis, topography and morphology codes, degree of spread.
National Death Index (NDI), 01/01/2002 - 31/12/2019	Death registrations and causes of death.	To calculate mortality rates for the cohort and censor individuals.	Date of death, underlying and contributing causes of death.
NSW Admitted Patient Data Collection (NSW APDC), 01/07/2001 - 30/06/2019; ACT Admitted Patient Collection (ACT APC), 01/07/2004 - 30/06/2018	Census of all inpatient episodes in all NSW/ACT public and private hospitals, public multi-purpose services and private day procedure centres.	To identify harms and risks associated with prescribed opioids, ascertain co-morbid diseases.	Dates of admission, separation and procedures, diagnostic and procedure codes, admission costs, separation mode, hospital type, hospital location.
NSW Emergency Department Data Collection (NSW EDDC), 01/01/2005 - 09/07/2019; ACT Emergency Department Data Collection (ACT	All visits to participating emergency departments in NSW/ACT.	To identify harms and risks associated with prescribed opioids.	Dates of presentation and separation, referral source, arrival mode, visit type, triage, diagnosis, separation mode.

© 2023 Gisev N et al. *JAMA Network Open.*

EDDC), 01/07/2005 - 02/07/2018			
**Dataset name and date range**	**Description of dataset**	**Purpose of dataset**	**Key variables of interest[a]**
NSW Controlled Drugs Data Collection (CoDDaC), 01/01/1985 - 31/12/2019.	Opioid substitution therapy (methadone/buprenorphine) treatment episodes in NSW. The data collection system for CoDDaC is the Electronic Recording and Reporting of Controlled Drugs (ERRCD), which was implemented in September 2016 to replace the legacy Pharmaceutical Drugs of Addiction System (PHDAS).	To identify individuals with a history of opioid dependence subsequently prescribed opioids; to examine risk of treatment for iatrogenic opioid dependence.	Treatment entry and exit dates, type of medicine authorised.
Mental Health Ambulatory Collection (MH-AMB), 01/01/2001 - 30/06/2018	Records on the assessment, treatment, rehabilitation or care of non-admitted mental health patients in NSW.	To identify individuals with mental health disorders and their treatment patterns.	Date of service, mental health diagnoses, services provided.

ACT: Australian Capital Territory; NSW: New South Wales
[a] Most collections hold patient demographics including age, sex

Note:

Although data from the NSW Emergency Department Data Collection (NSW EDDC), ACT Admitted Patient Collection (ACT APC) and ACT Emergency Department Data Collection (ACT EDDC) were included in the linkage to capture additional acute healthcare service use, information from these datasets were not included in establishing baseline characteristics of the cohort as the date ranges for these datasets do not extend back to the first date of observation for the cohort.

© 2023 Gisev N et al. *JAMA Network Open.*

eTable 3. ICD-10 and ATC Codes Used to Identify Medical Conditions of Interest

Condition	ICD-10 codes[a]	WHO ATC codes[b]
**Cardiovascular**		
Arrhythmia	I49.9	C01AA05, C01BA01-C01BD01
Congestive heart failure	I09.9, I11.0, I13.0, I13.2, I25.5, I42.0, I42.5-I42.9, I43, I50, P29.0	C03CA01-C03CC01
Hyperlipidaemia	E78.2- E78.5	C10AA01-C10BX03
Hypertension	I10, I11, I12, I13, I15	C03AA01-C03BA11, C03DA01-CO3AE01, C09BA02-C09BA09, C09DA02-C09DA07, C02AB01-C02AC05, C02DB02-C02KX01
Ischaemic heart disease	I20-I25	C01DA02-C01DA70, C01DX16, C08EX02
**Endocrine**		
Diabetes	E10, E11, E12, E13, E14	A10AA01–A10BX99
Hypothyroidism	E03.1, E03.8, E03.9	H03AA01-H03AA02
Hyperthyroidism	E05.10, E05.80, E05.90, E05.91	H03BB01
**Mental and Neurological**		
Anxiety	F40-F41, F43.22, F43.23	N05BA01–N05BA12, N05BE01
Dementia	F00-F03, F05.1, G30, G31.1	N06AA01–N06AG02, N06AX03–N06AX11, N06AX13–N06AX18, N06AX21–N06AX26
Depression	F20.4, F31.3-F31.5, F32, F33, F34.1, F41.2, F43.2	N06A
Parkinson's disease	G20, G21, G31.83, G90.3	N04AA01-N04BX03
Psychoses	F20, F22-F25, F28, F29, F30.2, F31.2, F31.5	N05AA01-N05AB02, N05AB06-N05AL07, N05AX01-N05AX17
Opioid use disorder	F11	N/A
Other substance use disorder	F12-F19, Z71.5, Z72.2	N07B except N07BC, N06AX12
**Musculoskeletal**		
Osteoporosis	M81.0, M81.6, M81.8, M83	H05, M05BA, M05BB
Rheumatic disease	M05, M06x, M31.5, M32 - M34, M35.1, M35.3, M36.0	A07EC, L01BA, L04AA, L04AX, M01C, P01BA, M01B, M03B, J01
**Respiratory**		
Asthma	J45, J46	R03BA, R03DC, R03AK
Chronic obstructive pulmonary disease	J40-J44	R03BB04, R03BB05, R03BB06, R03BB07, R03AC18, 8432T, 8519J, 10018G, 8750M, 10199T, R03AL04, R03AL03, R03AL06, R03AL05
**Other**		
Cancer	C00-C26, C30-C34, C37- C41, C43-C58, C60- C76, C81-C85, C88, C90-C97, Z51.1	L01AA01-L01AX04, L01BA01, L01BA03- L01XX53, L02BG03, L02BG04, L02BG06, L02BB01-L02BB04, L02BX01-

© 2023 Gisev N et al. *JAMA Network Open.*

		L02BX03, L04AX02, L04AX04, L04AX06, L02BA01, L02AE03, L02AE02
**Condition**	**ICD-10 codes[a]**	**ATC codes[b]**
Chronic liver failure	B18, K70.0 - K70.3, K70.9, K71.3 - K71.5, K71.7, K73, K74, K76.0, K76.2 - K76.4, K76.8, K76.9, Z94.4	A06AD11
Hepatitis C	Z22.50, Z22.52, Z22.59, Z86.19, B18.2, B19.20, B19.21, B17.8	J05AB54
Human immunodeficiency virus (HIV)/acquired immunodeficiency syndrome (AIDS)	B20-B22, B24	J05AE-J05AE08, J05AF01-J05AG03, J05AR, J05AX07
Renal disease	I12.0, I13.1, N03.2 - N03.7, N05.2 - N05.7, N18, N19, N25.0, Z49.0 - Z49.2, Z94.0, Z99.2	B03XA01-B03XA02, A11CC01-A11CC04, V03AE02

ATC: Anatomical Therapeutic Chemical classification; ICD-10: International Statistical Classification of Diseases and Related Health Problem 10th revision

[a] Source: World Health Organization. International Statistical Classification of Diseases and Related Health Problems, 10th Revision 2015. Available from: http://apps.who.int/classifications/icd10/browse/2015/en

[b] Source: World Health Organization Collaborating Centre for Drugs Statistics Methodology. ATC/DDD Index 2021. Available from: http://www.whocc.no/atc_ddd_index/

© 2023 Gisev N et al. *JAMA Network Open.*

eTable 4. MBS Item Codes for Primary Care, Allied Health, and Specialist Services

Service	MBS item codes
**GP visits**	
GP short visit	Item 3, 4, 2095, 90020
GP standard visit	Item 23, 24, 2144, 90035
GP long visit	Item 36, 37, 2180, 90043
GP prolonged visit	Item 44, 47, 2193, 90051
GP telehealth consultation	Subgroup A30.1, A30.2; Item 812, 827, 829, 867, 868, 869, 873, 876, 881, 885, 891, 892
GP Residential Aged Care Facility attendance	Group A35; Item 232, 249, 731, 772, 776, 788, 789, 829, 869, 881, 892, 903, 2125, 2138, 2179, 2220, 5010, 5028, 5049, 5067, 5260, 5263, 5265, 5267
GP after-hours after hours	Group A11, A22, A23; Subgroup A7.10; Item 597, 598, 585, 588, 591, 594, 599, 600, 733, 737, 741, 745, 761, 763, 766, 769, 772, 776, 788, 789, 5000, 5003, 5010, 5020, 5023, 5028, 5040, 5043, 5049, 5060, 5063, 5200, 5203, 5207, 5208, 5220, 5223, 5227, 5228, 5260, 5263, 5265, 5267
GP Acupuncture	Item 173, 193, 195, 197, 199
GP Focused Psychological Strategies	Group A6; Subgroup A20.2, A7.4; Item 283, 285, 286, 287, 371, 372
GP Health Assessment	Group A14; Subgroup A7.5
GP Chronic Disease Management plan	Subgroup A15.1; Item 229, 230, 231, 232, 233
GP Multidisciplinary Case Conference	Item 235, 236, 237, 238, 239, 240, 243, 244, 735, 739, 743, 747, 750, 758
GP Mental Health Plan	Subgroup A20.1; Item 272, 276, 277, 279, 281, 282, 894, 896, 898, 2121, 2150 and 2196
Other non-referred medical practitioner attendances	Group A2, A16; Subgroup A7.2, A35.3, A35.4; Item 899, 901, 905, 906, 90002
**Allied health practitioner visits**	
Chiropractor	Item 10964, 81345
Exercise physiologist	Item 10953, 81110, 81115, 81315
Osteopath	Item 10966, 81350
Physiotherapist	Item 10960, 81335
Podiatrist	Item 10962, 81340
Psychologist	Group M6, Item 80001, 80011, 80021, 10968, 80100, 80101, 80105, 80110, 80111, 80115, 80120, 80121, 81355, 82000, 82015

GP: General practitioner; MBS: Medical Benefits Scheme

eTable 5. ATC Codes for Non-opioid Analgesic and Psychotropic Medicines

Medicine	ATC code[a]
**Non-opioid analgesic medicines**	
Paracetamol	N02BE01
Pregabalin	N03AX16<br>Or N02BG [b] + PBS item code (02335X, 02348N, 02355Y or 02363J)
Gabapentin	N03AX12<br>Or N02BG except pregabalin[b]
Triptans	N02CC
Pizotifen	N02CX01
**Non-selective Non-steroidal anti-inflammatory drugs (NSAIDs)**	
Diclofenac	M01AB05
Ibuprofen	M01AE01
Indomethacin	M01AB01
Ketoprofen	M01AE03
Mefenamic Acid	M01AG01
Naproxen	M01AE02
Piroxicam	M01AC01
Sulindac	M01AB02
**Selective Cox-2 inhibitors**	
Celecoxib	M01AH01
Lumiracoxib	M01AH06
Meloxicam	M01AC06
Rofecoxib	M01AH02
**Psychotropic medicines**	
Antiepileptics	N03A except pregabalin and gabapentin
Antipsychotics	N05A (includes lithium N06AX[b])
Anxiolytics	N05B
Hypnotics and sedatives	N05C
Antidepressants	N06A

ATC: Anatomical Therapeutic Chemical classification
[a] Source: World Health Organization Collaborating Centre for Drugs Statistics Methodology. ATC/DDD Index 2021. Available from: http://www.whocc.no/atc_ddd_index/
[b] Modified ATC code used in the PBS dataset

© 2023 Gisev N et al. *JAMA Network Open.*

eTable 6. Model Selection - BIC of Models With Varying Number of Trajectory Groups and Polynomial Order

	Number of Trajectory Groups						
**Polynomial Order**	**1**	**2**	**3**	**4**	**5**	**6**	**7**
**1**	7,691,320						
**2**	6,284,548	5,218,298	5,147,691				
**3**	6,278,808	5,214,280	5,141,951	5,031,893	4,958,201		
**4**	6,274,790	5,209,879	5,136,624	5,030,678	4,956,507	4,911,906	4,894,329

BIC: Bayesian Information Criterion

© 2023 Gisev N et al. *JAMA Network Open.*

eFigure 2. Model selection - Trajectory Plots of Models With Varying Opioid Use Groups

a) 3-trajectory group model

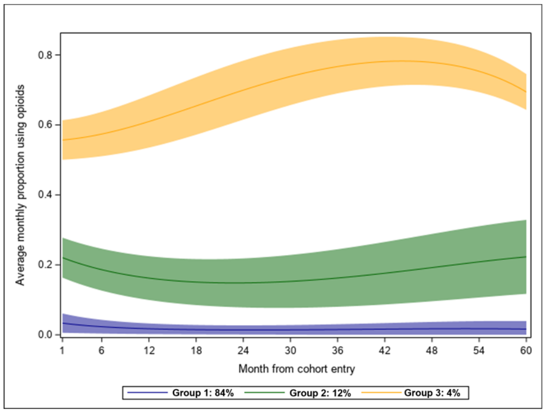

b) 4-trajectory group model

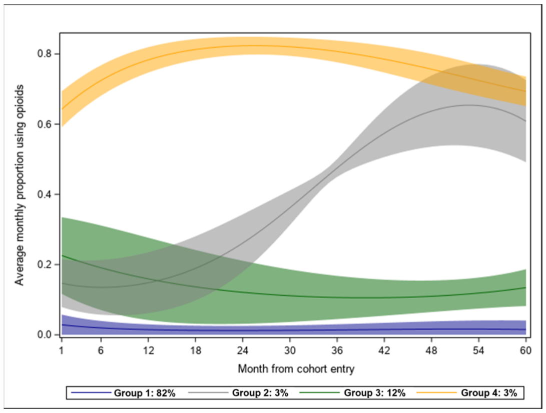

c) 6-trajectory group model

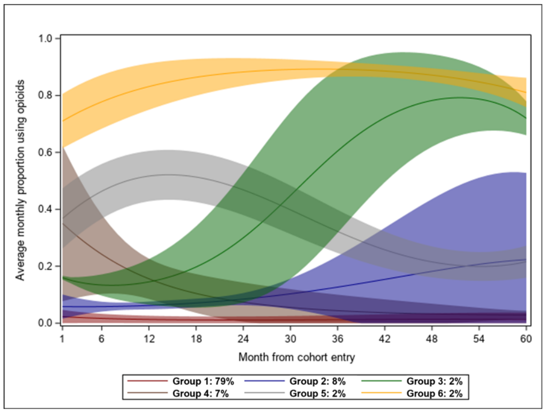

© 2023 Gisev N et al. *JAMA Network Open.*

eFigure 3. Heat Map Showing the Relative Prevalence of Baseline Characteristics by Trajectory Group

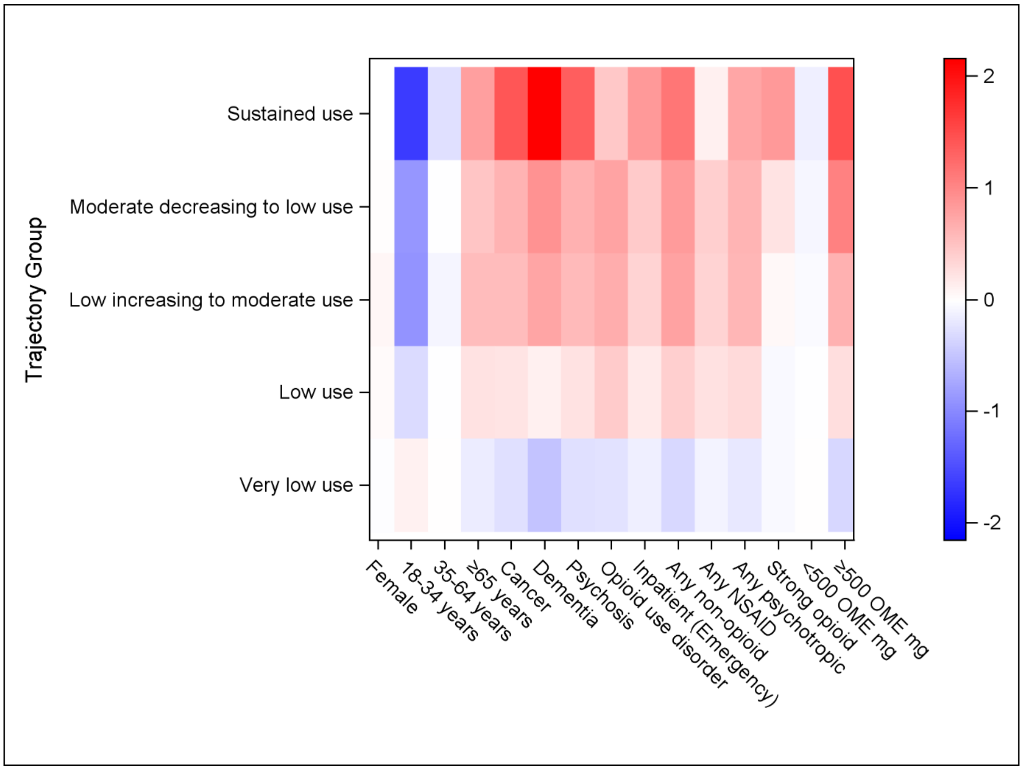

NSAID: Non-steroidal anti-inflammatory drug; OME mg: oral morphine equivalent milligrams

Key: This heat map summarises the relative prevalence of selected baseline characteristics (sociodemographics, clinical characteristics, medicine use, and health services utilisation) by opioid use trajectory group. The prevalence of each characteristic within each trajectory group relative to the entire cohort is displayed in colour scale, ranging from blue (lower prevalence) to white (cohort average prevalence), and red (higher prevalence). Characteristics which are more underrepresented within a trajectory group (relative to the entire cohort) are shown using areas with a greater intensity of blue, and characteristics which are more overrepresented within a trajectory group are shown using areas with a greater intensity of red.

© 2023 Gisev N et al. *JAMA Network Open.*

eFigure 4. Overlaid Histograms of Age by Trajectory Group

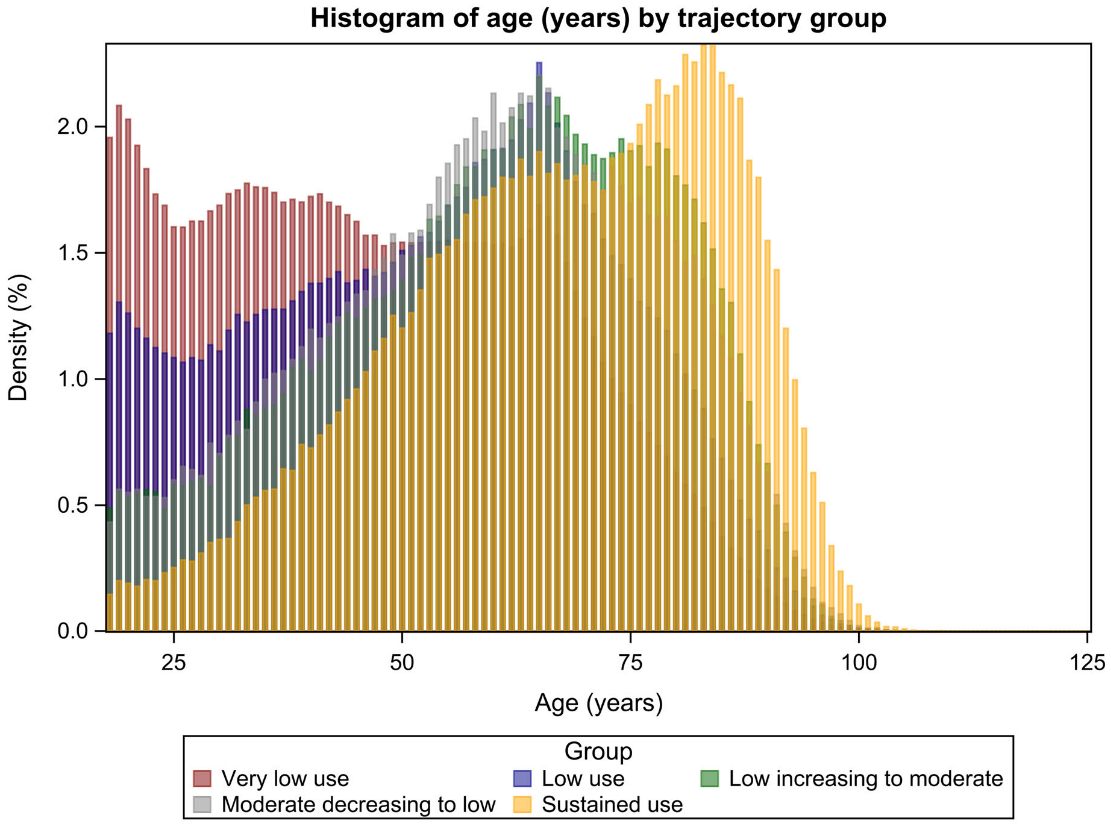

© 2023 Gisev N et al. *JAMA Network Open.*

eTable 7. Comparison of Trajectory Group Membership for the Main and Sensitivity Analyses

	**Main analysis**			**Sensitivity analysis**		
				**% of study cohort in each trajectory group**		
**Trajectory Group**	**% of study cohort in each trajectory group**	**% of trajectory group with <5 years of follow-up for any reason**	**% of trajectory group who died during follow-up**	**Excluding general beneficiaries prior to July 2013**	**Excluding people entering the cohort from 2014**	**Excluding all people with <5 years of follow-up for any reason**
	**N= 3,474,490**			**N=3,207,459**	**N=2,164,524**	**N=1,923,364**
Very low use	74	48	4	73	69	72
Low use	18	33	10	18	20	20
Low increasing to moderate use	3	37	21	3	4	3
Moderate decreasing to low use	3	34	20	3	4	3
Sustained use	3	62	50	3	4	2
Cohen's Kappa*	-	-	-	0.99	0.93	0.96

* Agreement between individual group allocations in the main analysis and sensitivity analyses

© 2023 Gisev N et al. *JAMA Network Open.*

eFigure 5. Trajectory Plots for Sensitivity Analyses

a) Excluding general beneficiaries 2003-2013

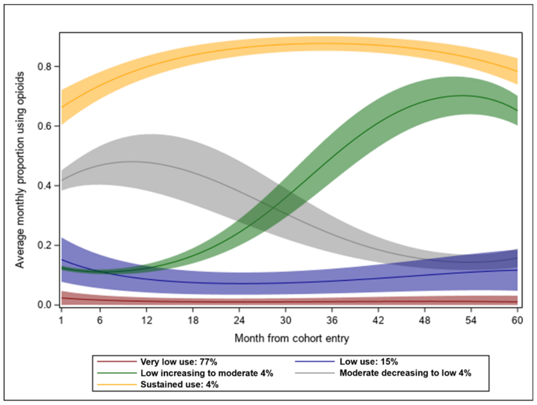

b) Excluding people entering the cohort after 2014

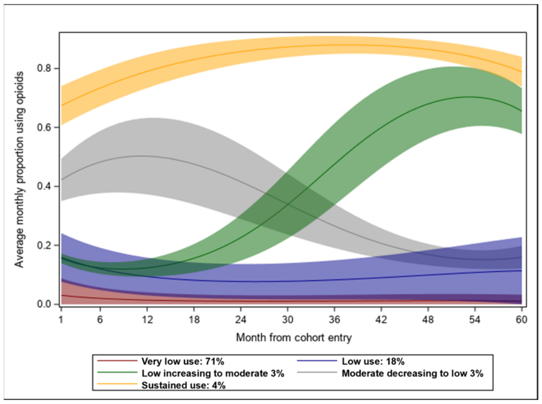

c) Excluding people with <5 years of follow-up for any reason

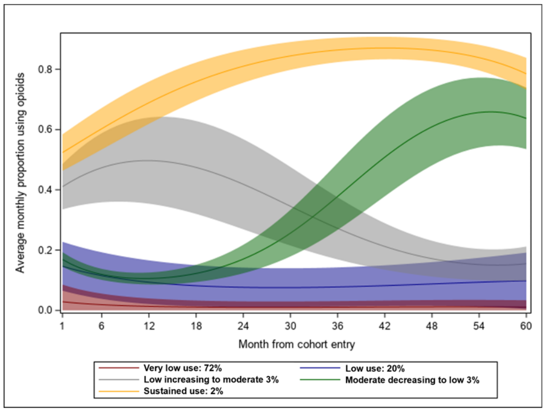

© 2023 Gisev N et al. *JAMA Network Open.*
