# Supplementary material for: Five-Year Trajectories of Prescription Opioid Use
Source: JAMA Netw Open. 2023 Aug 10;6(8):e2328159. doi: 10.1001/jamanetworkopen.2023.28159 (PMC10415961; doi:10.1001/jamanetworkopen.2023.28159)
Supplement: Supplement 2. — Data Sharing Statement [file jamanetwopen-e2328159-s002.pdf]

Data Sharing Statement

Gisev. Five-Year Trajectories of Prescription Opioid Use. *JAMA Netw Open*. Published August 10, 2023. doi:10.1001/jamanetworkopen.2023.28159

Data
Data available: No

Additional Information
Explanation for why data not available: This study used de-identified person-level data from the POPPY II study. The data are not publicly available due to ethical restrictions.
